# Supplementary material for: MetaRibo-Seq measures translation in microbiomes
Source: Nat Commun. 2020 Jun 29;11:3268. doi: 10.1038/s41467-020-17081-z (PMC7324362; doi:10.1038/s41467-020-17081-z)
Supplement: Supplementary file 10 — Supplementary Data 7 [file 41467_2020_17081_MOESM10_ESM.zip › File2/Confidence_High_Taxonomy/377241_out.krona.html]

Javascript must be enabled to view this page.

members
magnitude
magnitudeUnassigned
count
unassigned
taxon
rank

377241\_out

339

339
2
superkingdom

339
phylum
976

339
200643
class

339
171549
order

2005519
family
1

1
1348911
genus

1099853

SRS019397\_contig\_number\_9175
species
1

family
171552
2

1
1926672
genus

1
1852370

SRS143070\_contig\_number\_1457
species

577309
genus
1


SRS023914\_contig\_number\_3422
454155
species
1

family
815
336

12
816
336

SRS015264\_contig\_number\_12276SRS018351\_contig\_number\_16427SRS018427\_contig\_number\_27298SRS019030\_contig\_number\_16973SRS019910\_contig\_number\_25414SRS043667\_contig\_number\_6316SRS048870\_contig\_number\_4444SRS049959\_contig\_number\_52434SRS050299\_contig\_number\_7589SRS054352\_contig\_number\_23387SRS077086\_contig\_number\_5238SRS893300\_contig\_number\_7760
genus

species
2292944

SRS024625\_contig\_number\_1094
1

1
species

SRS050520\_contig\_number\_71
1638779

species

SRS058723\_contig\_number\_1512SRS1054928\_contig\_number\_2761
817
2

2

SRS105082\_contig\_number\_13219SRS893270\_contig\_number\_14781
338188
species

2
species

SRS893358\_contig\_number\_13728SRS893378\_contig\_number\_14554
469593


SRS140492\_contig\_number\_4107
820
species
1

1
371601

SRS011405\_contig\_number\_11766
species

5
species
671267

SRS052027\_contig\_number\_8744SRS098073\_contig\_number\_16477SRS148721\_contig\_number\_17369SRS149325\_contig\_number\_14832SRS893256\_contig\_number\_1624

283
species

SRS011084\_contig\_number\_3192SRS011239\_contig\_number\_25687SRS011302\_contig\_number\_23070SRS012902\_contig\_number\_46SRS013158\_contig\_number\_536SRS013382\_contig\_number\_323SRS013521\_contig\_number\_8846SRS013687\_contig\_number\_32658SRS013965\_contig\_number\_37196SRS014235\_contig\_number\_3783SRS014287\_contig\_number\_18269SRS014313\_contig\_number\_1013SRS014412\_contig\_number\_20869SRS014459\_contig\_number\_34712SRS014534\_contig\_number\_141SRS014683\_contig\_number\_19154SRS014736\_contig\_number\_7342SRS014855\_contig\_number\_5177SRS014948\_contig\_number\_15370SRS014979\_contig\_number\_36863SRS015065\_contig\_number\_33908SRS015190\_contig\_number\_2870SRS015217\_contig\_number\_14564SRS015486\_contig\_number\_942SRS015578\_contig\_number\_17425SRS015663\_contig\_number\_25101SRS015694\_contig\_number\_15945SRS015782\_contig\_number\_13097SRS015854\_contig\_number\_13723SRS015960\_contig\_number\_21015SRS016056\_contig\_number\_1655SRS016095\_contig\_number\_21858SRS016132\_contig\_number\_947SRS016203\_contig\_number\_8792SRS016335\_contig\_number\_18779SRS016495\_contig\_number\_contig-100\_374.118483SRS016629\_contig\_number\_1359SRS016753\_contig\_number\_3968SRS016840\_contig\_number\_2202SRS016954\_contig\_number\_11216SRS017103\_contig\_number\_34925SRS017307\_contig\_number\_18026SRS017375\_contig\_number\_244SRS017433\_contig\_number\_10279SRS017521\_contig\_number\_16177SRS017859\_contig\_number\_1041SRS018623\_contig\_number\_15118SRS018817\_contig\_number\_10423SRS018836\_contig\_number\_2747SRS018888\_contig\_number\_10011SRS018936\_contig\_number\_12137SRS019068\_contig\_number\_3536SRS019161\_contig\_number\_22378SRS019267\_contig\_number\_1474SRS019445\_contig\_number\_10040SRS019496\_contig\_number\_9882SRS019601\_contig\_number\_14658SRS019693\_contig\_number\_2486SRS019808\_contig\_number\_21068SRS019968\_contig\_number\_8793SRS020233\_contig\_number\_47358SRS020328\_contig\_number\_19058SRS020394\_contig\_number\_10655SRS020508\_contig\_number\_166SRS020622\_contig\_number\_1998SRS020869\_contig\_number\_34522SRS021153\_contig\_number\_3725SRS021219\_contig\_number\_11965SRS021484\_contig\_number\_15277SRS021948\_contig\_number\_1805SRS022071\_contig\_number\_23905SRS022609\_contig\_number\_15680SRS022713\_contig\_number\_236SRS023526\_contig\_number\_10113SRS023829\_contig\_number\_638SRS024009\_contig\_number\_9126SRS024075\_contig\_number\_9744SRS024492\_contig\_number\_7318SRS042284\_contig\_number\_4176SRS042966\_contig\_number\_14504SRS043411\_contig\_number\_9092SRS043841\_contig\_number\_2169SRS044535\_contig\_number\_21526SRS045244\_contig\_number\_4288SRS045645\_contig\_number\_14744SRS045739\_contig\_number\_1385SRS045826\_contig\_number\_19090SRS046068\_contig\_number\_2636SRS046369\_contig\_number\_12515SRS046502\_contig\_number\_4724SRS046810\_contig\_number\_contig-100\_20.21SRS047014\_contig\_number\_37236SRS047044\_contig\_number\_13374SRS047433\_contig\_number\_18997SRS048164\_contig\_number\_20657SRS049402\_contig\_number\_11176SRS049446\_contig\_number\_2820SRS049712\_contig\_number\_contig-100\_439.130213SRS049773\_contig\_number\_8553SRS049896\_contig\_number\_22411SRS049900\_contig\_number\_11023SRS049995\_contig\_number\_22945SRS050026\_contig\_number\_4131SRS050422\_contig\_number\_36031SRS050752\_contig\_number\_2408SRS050801\_contig\_number\_3023SRS050941\_contig\_number\_20138SRS050974\_contig\_number\_1818SRS050998\_contig\_number\_16543SRS051610\_contig\_number\_11496SRS051882\_contig\_number\_11731SRS052697\_contig\_number\_26831SRS053087\_contig\_number\_1839SRS053214\_contig\_number\_13994SRS053335\_contig\_number\_14187SRS053398\_contig\_number\_23952SRS054059\_contig\_number\_5149SRS054905\_contig\_number\_23310SRS055966\_contig\_number\_620SRS056259\_contig\_number\_14013SRS056273\_contig\_number\_14059SRS056537\_contig\_number\_8193SRS057478\_contig\_number\_2075SRS057480\_contig\_number\_6322SRS058268\_contig\_number\_17SRS062427\_contig\_number\_9468SRS062654\_contig\_number\_1078SRS062701\_contig\_number\_5117SRS063127\_contig\_number\_2651SRS063190\_contig\_number\_9650SRS063370\_contig\_number\_9993SRS063489\_contig\_number\_5582SRS063518\_contig\_number\_36952SRS063813\_contig\_number\_4785SRS063928\_contig\_number\_contig-100\_25.12861SRS064557\_contig\_number\_5197SRS064645\_contig\_number\_1018SRS064973\_contig\_number\_694SRS065176\_contig\_number\_10507SRS065397\_contig\_number\_4575SRS065504\_contig\_number\_27864SRS074586\_contig\_number\_4753SRS074670\_contig\_number\_2482SRS074964\_contig\_number\_2050SRS075078\_contig\_number\_19620SRS075398\_contig\_number\_2632SRS075716\_contig\_number\_2201SRS075773\_contig\_number\_10196SRS075821\_contig\_number\_15511SRS075984\_contig\_number\_12194SRS076756\_contig\_number\_3146SRS076876\_contig\_number\_565SRS076929\_contig\_number\_17609SRS076976\_contig\_number\_20649SRS077194\_contig\_number\_14618SRS077294\_contig\_number\_14582SRS077335\_contig\_number\_9098SRS077454\_contig\_number\_3064SRS077502\_contig\_number\_12806SRS077552\_contig\_number\_4267SRS077589\_contig\_number\_7924SRS077730\_contig\_number\_15953SRS077991\_contig\_number\_418SRS078176\_contig\_number\_1861SRS078665\_contig\_number\_1625SRS097920\_contig\_number\_12798SRS097958\_contig\_number\_1609SRS098061\_contig\_number\_8105SRS098571\_contig\_number\_9887SRS098644\_contig\_number\_28749SRS098827\_contig\_number\_219SRS098881\_contig\_number\_7001SRS100021\_contig\_number\_445SRS100907\_contig\_number\_8671SRS101376\_contig\_number\_21715SRS104036\_contig\_number\_6873SRS104084\_contig\_number\_12845SRS1041031\_contig\_number\_28958SRS1041036\_contig\_number\_1395SRS1041037\_contig\_number\_14016SRS1041039\_contig\_number\_20319SRS1041090\_contig\_number\_5221SRS1041091\_contig\_number\_20196SRS1041092\_contig\_number\_247SRS1041095\_contig\_number\_3107SRS1041116\_contig\_number\_9102SRS1041118\_contig\_number\_10870SRS1041129\_contig\_number\_5346SRS1041130\_contig\_number\_1986SRS1041133\_contig\_number\_27424SRS1041137\_contig\_number\_31397SRS1041138\_contig\_number\_9617SRS1041140\_contig\_number\_19909SRS1041141\_contig\_number\_8138SRS1041142\_contig\_number\_8598SRS1041143\_contig\_number\_3490SRS1041144\_contig\_number\_3238SRS1041147\_contig\_number\_2303SRS1041157\_contig\_number\_5461SRS1041164\_contig\_number\_719SRS104327\_contig\_number\_6271SRS104400\_contig\_number\_14327SRS104485\_contig\_number\_88SRS104693\_contig\_number\_5148SRS104975\_contig\_number\_8473SRS1054716\_contig\_number\_11698SRS1055022\_contig\_number\_7523SRS1055038\_contig\_number\_5336SRS1055049\_contig\_number\_12352SRS1055056\_contig\_number\_12132SRS1055069\_contig\_number\_2035SRS1055076\_contig\_number\_12366SRS1055083\_contig\_number\_145SRS1055095\_contig\_number\_586SRS140513\_contig\_number\_5277SRS142503\_contig\_number\_16966SRS142542\_contig\_number\_21655SRS142599\_contig\_number\_18037SRS142890\_contig\_number\_38621SRS142923\_contig\_number\_19652SRS142980\_contig\_number\_1632SRS143085\_contig\_number\_2125SRS143148\_contig\_number\_contig-100\_124.145903SRS143181\_contig\_number\_14318SRS143342\_contig\_number\_9759SRS143372\_contig\_number\_7048SRS143417\_contig\_number\_3417SRS143722\_contig\_number\_11963SRS143780\_contig\_number\_3970SRS143876\_contig\_number\_12964SRS143895\_contig\_number\_37668SRS143991\_contig\_number\_20151SRS144183\_contig\_number\_39946SRS144362\_contig\_number\_6677SRS144506\_contig\_number\_5911SRS144537\_contig\_number\_41823SRS144603\_contig\_number\_15221SRS144714\_contig\_number\_27676SRS144753\_contig\_number\_12240SRS145308\_contig\_number\_1153SRS145497\_contig\_number\_1517SRS146764\_contig\_number\_23957SRS146812\_contig\_number\_24577SRS146813\_contig\_number\_11825SRS147022\_contig\_number\_154SRS147039\_contig\_number\_1664SRS147271\_contig\_number\_5943SRS147346\_contig\_number\_8518SRS147377\_contig\_number\_3738SRS147425\_contig\_number\_8218SRS147557\_contig\_number\_33379SRS147614\_contig\_number\_7237SRS147766\_contig\_number\_4455SRS147977\_contig\_number\_3618SRS148091\_contig\_number\_11473SRS148196\_contig\_number\_40665SRS148253\_contig\_number\_7328SRS148424\_contig\_number\_28577SRS148784\_contig\_number\_27360SRS148817\_contig\_number\_13171SRS148970\_contig\_number\_1688SRS149075\_contig\_number\_7522SRS149181\_contig\_number\_16630SRS149244\_contig\_number\_5052SRS892882\_contig\_number\_6915SRS893170\_contig\_number\_9536SRS893172\_contig\_number\_5302SRS893187\_contig\_number\_18507SRS893252\_contig\_number\_17303SRS893253\_contig\_number\_49SRS893259\_contig\_number\_1573SRS893279\_contig\_number\_11701SRS893285\_contig\_number\_619SRS893292\_contig\_number\_7322SRS893295\_contig\_number\_13128SRS893327\_contig\_number\_7919SRS893341\_contig\_number\_4307SRS893366\_contig\_number\_23820SRS893369\_contig\_number\_5102SRS893373\_contig\_number\_27125SRS893380\_contig\_number\_1416SRS893383\_contig\_number\_32789SRS971275\_contig\_number\_39089
821

species
1263040

SRS055017\_contig\_number\_28149
1

3
species

SRS017247\_contig\_number\_217SRS019381\_contig\_number\_5259SRS097889\_contig\_number\_32543
818

1
457395

SRS017745\_contig\_number\_681
species

species
310297

SRS078242\_contig\_number\_21750
1

2

SRS016897\_contig\_number\_63SRS077641\_contig\_number\_5686
329854
species

1
1739298

SRS052576\_contig\_number\_107
species

2

SRS051031\_contig\_number\_6358SRS893382\_contig\_number\_1928
357276
species

1
species
28116

SRS016989\_contig\_number\_3671

1
species

SRS011134\_contig\_number\_43723
2184759


SRS013638\_contig\_number\_8021SRS019178\_contig\_number\_4515SRS023971\_contig\_number\_657SRS045004\_contig\_number\_3157SRS077231\_contig\_number\_24681SRS1041139\_contig\_number\_350SRS144297\_contig\_number\_4654SRS893288\_contig\_number\_300
46506
species
8

4

SRS012969\_contig\_number\_20257SRS077849\_contig\_number\_12286SRS103987\_contig\_number\_2356SRS142781\_contig\_number\_551
47678
species


SRS017191\_contig\_number\_17446
2292935
species
1
